# The development of laying hen locomotion in 3D space is affected by early environmental complexity and genetic strain

**DOI:** 10.1038/s41598-023-35956-1

**Published:** 2023-06-21

**Authors:** Ana K. Rentsch, Erin Ross, Alexandra Harlander, Lee Niel, Janice M. Siegford, Tina M. Widowski

**Affiliations:** 1 Department of Animal Biosciences, University of Guelph, Guelph, ON Canada; 2 Department of Population Medicine, University of Guelph, Guelph, ON Canada; 3 Department of Animal Science, Michigan State University, East Lansing, MI USA

**Keywords:** Developmental biology, Ecology, Evolution, Genetics, Zoology

## Abstract

Adult laying hens are increasingly housed in spatially complex systems, e.g., non-cage aviaries, where locomotion between elevated structures can be challenging for these gallinaceous birds. This study assessed the effect of early environmental complexity on spatial skills in two genetic strains. Brown (B) and white (W) feathered birds were raised in: Conventional cages with minimal complexity (*Conv*) or rearing aviaries with low (*Low*), intermediate (*Mid*), or high complexity (*High*). Birds from each housing treatment were challenged at three different time points in three different, age-appropriate vertical spatial tasks. Whites performed better than brown birds in all tests regardless of rearing environment. In chicks, test performance was predominantly explained by variation between replicates and differences in motivation for test participation. Treatment effects were seen in pubertal birds (pullets), with pullets from aviaries performing better than those from *Conv*. White *High* pullets performed better than white *Mid* or *Low*, an effect that was not found in browns. Pullets preferred to use a ramp to move downwards, but only when ramps had previously been experienced and when the ramp was not too steep. Overall, early environmental complexity affected spatial skills of laying hen pullets with stronger effects in white than brown feathered birds.

## Introduction

Laying hens (egg-laying chickens) housed in non-cage aviary systems can perform a wide range of behaviours, including species-specific behavioural needs such as perching, dustbathing, foraging, and pre-laying behaviour^1,2^. At the same time, three-dimensional (3D) complexity and large group sizes increase the risk of injury through falls and collisions^3–5^. In a study conducted in commercial aviary housing with a white feathered (Lohmann) strain, video observations showed that 9–21% of vertical transitions fail^6^. For successful locomotion in 3D space, physical and cognitive spatial skills are required^7^, and these skills are best learned when young^8^. It has been proposed that laying hen chicks perform the physical process of jumping without much difficulty and that cognitive skills are the ones that need developing to ensure success^9^.

In many species, such as fish^10–12^, rats^13,14^, and birds^15,16^, the effect of increased early life environmental complexity on brain morphology with potential positive effects on cognition has been well documented. Evidence suggests that there are sensitive periods during which the effect of environmental complexity has the greatest effect on brain and behavioural development^17,18^. Young pheasants reared with elevated perches during the first seven weeks of life, for instance, had better spatial skills shown by roosting more and higher and had improved spatial memory, making fewer mistakes in a radial maze compared to controls^19^. The study of the effect of early life experience on laying hen development is relatively new and under-researched^20^.

When specifically looking at laying hens, greater rearing complexity improved spatial abilities after transfer to aviaries at the beginning of lay^21^, with floor-raised hens using the upper aviary levels less than aviary-reared hens and showing lower accuracy in long flights and jumps. Appleby and Duncan^9^ proposed an early sensitive period where the use of elevated structures must be learned. Rearing with ramps improved^22,23^ and advanced the use of elevated structures in laying hen pullets^23^, suggesting more rapid development of spatial skills. Hens that had been reared with ramps showed less hesitation to use elevated tiers^22^ and ramps^23^ during lay. This indicates that enabling 3D space use during development could positively impact the learning process needed to appropriately navigate 3D space later in life.

The effect of early life complexity on spatial skills might be dependent on behavioural tendencies of particular genetic strains. Commercially used laying hen strains can be phylogenetically grouped into ‘brown’ and ‘white’ lineages based on eggshell and feather color^24^. Differences between brown and white feathered genetics have been documented for multiple physiological and behavioural traits^25,26^. Brown feathered strains have been shown to be less active, explore less, and be less motivated for social contact or foraging opportunities^27^. Differences in space use between brown and white laying hens have been shown in commercial^28^, commercial-like^28–30^, and experimental^31^ settings. White feathered pullets performed more aerial transitions and generally locomoted more in rearing aviaries than brown pullets^28^, potentially due to their proportionally larger keel bones and breast muscles and smaller leg muscles compared to brown layers^28,32^. Furthermore, it has been shown that laying hens of brown feathered genetic strains are better at successfully moving upwards than downwards between elevated perches^33,34^. Browns also refused to jump or had to balance at landing more often when completing downwards movements compared to moving upwards^34^.

This study aims to answer whether the degree of early life complexity and pullets’ genetic strain affect the development of spatial skills in 3D space, specifically vertical locomotion abilities and locomotory strategies. Brown and white pullets were raised in either conventional cages (minimal complexity, *Conv*) or one of three styles of rearing aviaries that differed in their spatial complexity (*Low*, *Mid*, *High*). Three different age-appropriate tests were used to assess spatial skills in week 6 (hurdle jumping for social re-instatement), week 16 (vertical navigation for food reward), and week 17 (vertical navigation with optional ramp use). We hypothesised that (1) performance in all spatial tests would improve with increasing rearing complexity (*High* > *Mid* > *Low* > *Conv*), (2) white feathered pullets would perform better than brown feathered pullets in all spatial tests, (3) whites would prefer aerial locomotion and browns would prefer ramp use, and (4) that *Conv* pullets with no prior ramp experience would be less likely to choose the ramp than aviary-reared pullets.

## Methods

Detailed methods are described in the supplementary material available in the online version at 10.1038/s41598-023-35956-1.

### Ethical approval

Animal use was approved, and its ethicality considered by the animal care committee of the University of Guelph (Animal Utilisation Protocol Nr 4127). All methods were performed in accordance with the Ontario regulation (https://www.ontario.ca/laws/regulations) and the Canadian Council on Animal Care (CCAC) guidelines for the ethical care of all animals used for scientific purposes (https://ccac.ca/en/guidelines-and-policies/the-guidelines/general-guidelines.html). No invasive interventions were performed apart from standard management procedures (e.g., vaccinations).

### Animals and management

A two by four factorial experiment was repeated four times with genetic strain (2 levels) and housing (4 levels) as the main effects. Four consecutive flocks of 1,497 one-day-old chicks each were delivered from the hatchery (Archer’s Poultry Farm Ltd., Ontario Canada) and divided between housing treatment groups. Each flock comprised two genetic strains that were housed separately: Lohmann Brown lite (brown) and Lohmann Selected Leghorn lite (white).

### Housing

**Figure 1:**
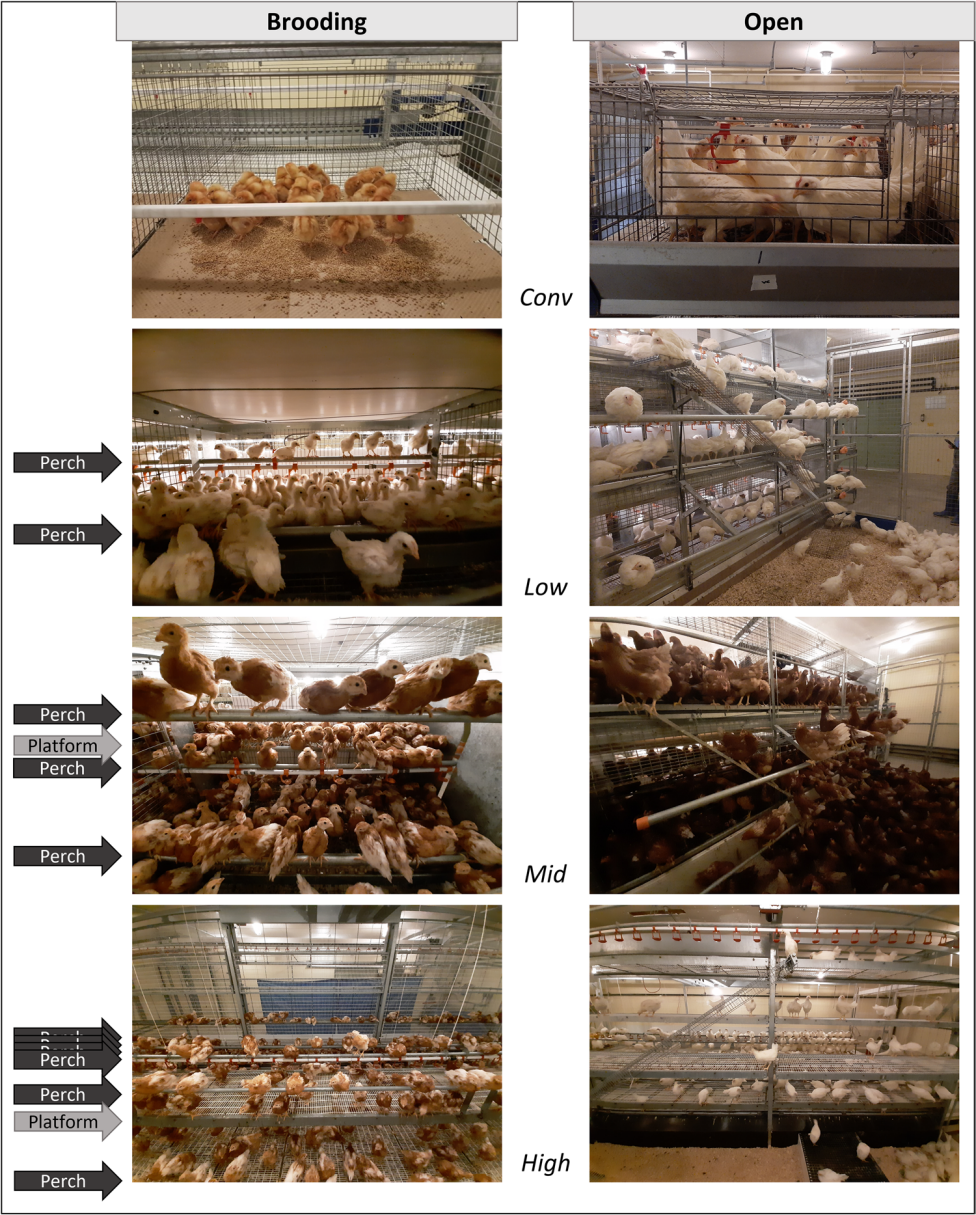
Photos of the different housing systems. The left side illustrates the brooding period (first six weeks) and the right column shows the open period (six-17 weeks). The location of perches and platforms are indicated for the brooding period only. Environmental complexity increased from top to bottom. The Figure was created using Microsoft PowerPoint for Microsoft 365 MSO version 2304.

The different housing treatments consisted of three styles of rearing aviaries incrementally increasing in environmental complexity (*Low*, *Mid*, and *High*) and conventional pullet cages (*Conv*). The rearing aviary differed most during the brooding phase (first 6 weeks) when chicks are typically confined to the brooding compartment. After the brooding phase, all aviaries were opened, and chicks gained access to a litter area, multiple elevated tiers, perches, and ramps (Fig. 1). *Low* had three brooding compartments, each furnished with two elevated perches (n = 345 per strain and flock). *Mid* also had three brooding compartments; each offered three elevated perches as well as an elevated platform (n = 432 per strain and flock). *High* had one brooding compartment that spanned the whole length of the barn and offered 6 elevated perches and an elevated platform (n = 600 birds per strain and flock). *Conv* were eight barren cages with minimal environmental complexity (n = 120 per strain and flock). The housing systems are described and visualised in more detail in Rentsch^35^ and Ross^36^. In weeks 6 and 16 of rearing, samples of birds were selected from these populations to be tested in three spatial tests. The sample size (n = 8/housing treatment) was largely dictated by logistic parameters (infrastructure and time). However, enough individuals were used in each test to satisfy the degrees of freedom needed to analyse population variation^37,38^ (see Supplementary Tables 1, 3, and 5 for detailed calculations).

### Hurdle test

**Figure 2:**
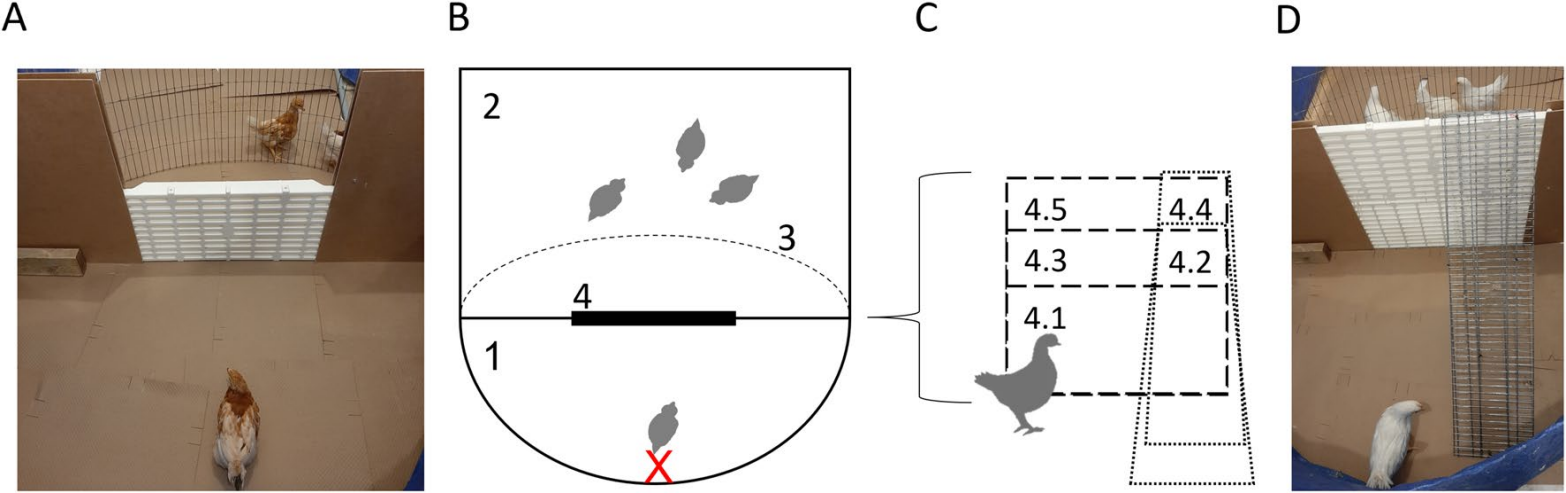
(**A**) A photo of a brown chick being tested in set up 4.1 (lowest hurdle). (**B**) Top view of the testing arena (200 × 170 cm). Section ‘1’ contained the test bird, the start location is indicated by the red X (70–80 cm from the hurdle). Section ‘2’ contained the group of birds intended for social incentive (120 × 170 cm). A wire fence ‘3’ (45 cm from the hurdle at the farthest point) kept the chicks in the social group from jumping into the testing section. ‘1’ and ‘2’ were divided by a solid wall with the plastic grid hurdle ‘4’ (55 cm long) in the centre. (**C)** Front view of the hurdles with different difficulty levels; ‘4.1’ 30 cm tall, ‘4.2’ 45 cm + 90 cm ramp, ‘4.3’ 45 cm tall, ‘4.4’ 60 cm + 90 cm ramp, and ‘4.5’ 60 cm tall. (**D)** A photo of a white chick being tested in set up 4.4 (tallest hurdle + ramp). Schematics were created using Microsoft PowerPoint for Microsoft 365 MSO version 2304.

In week six, a total of 1470 naïve chicks were selected as focal birds from the four flocks and subjected to a hurdle test. Two-hundred-and-ninety-four chicks were tested in each of the five test set-ups (Fig. 2C) that related to five different tasks or difficulty levels: three different hurdle heights (see Fig. 2A) and two ramp and hurdle combinations (see Fig. 2D) (see Supplementary Table 2). Before each test, three chicks from the same housing group as the focal chicks were placed on the far side of the hurdle along with some feed. Their purpose was to motivate focal chicks to cross the hurdle for social reinstatement^39^. To start a test, the focal chick was placed in the testing section (centre and opposite the hurdle, see Fig. 2B), facing the hurdle. The test lasted for 120 s or until the chick succeeded in crossing the hurdle. Behaviours measured were vocalisation bouts (1 ‘bout’ = 1–5 chirps with a natural gap for breathing between bouts), latency to start walking (‘walking’ = min three consecutive steps), the number of jumping attempts made (‘jump attempt’ = chick’s whole body lifts with both feet off the floor while facing the hurdle), crossing success (‘success’ = chick crosses or reaches top of hurdle with both feet), and strategy (crossing by ramp = 1, by jumping = 0).

### Vertical navigation test

**Figure 3:**
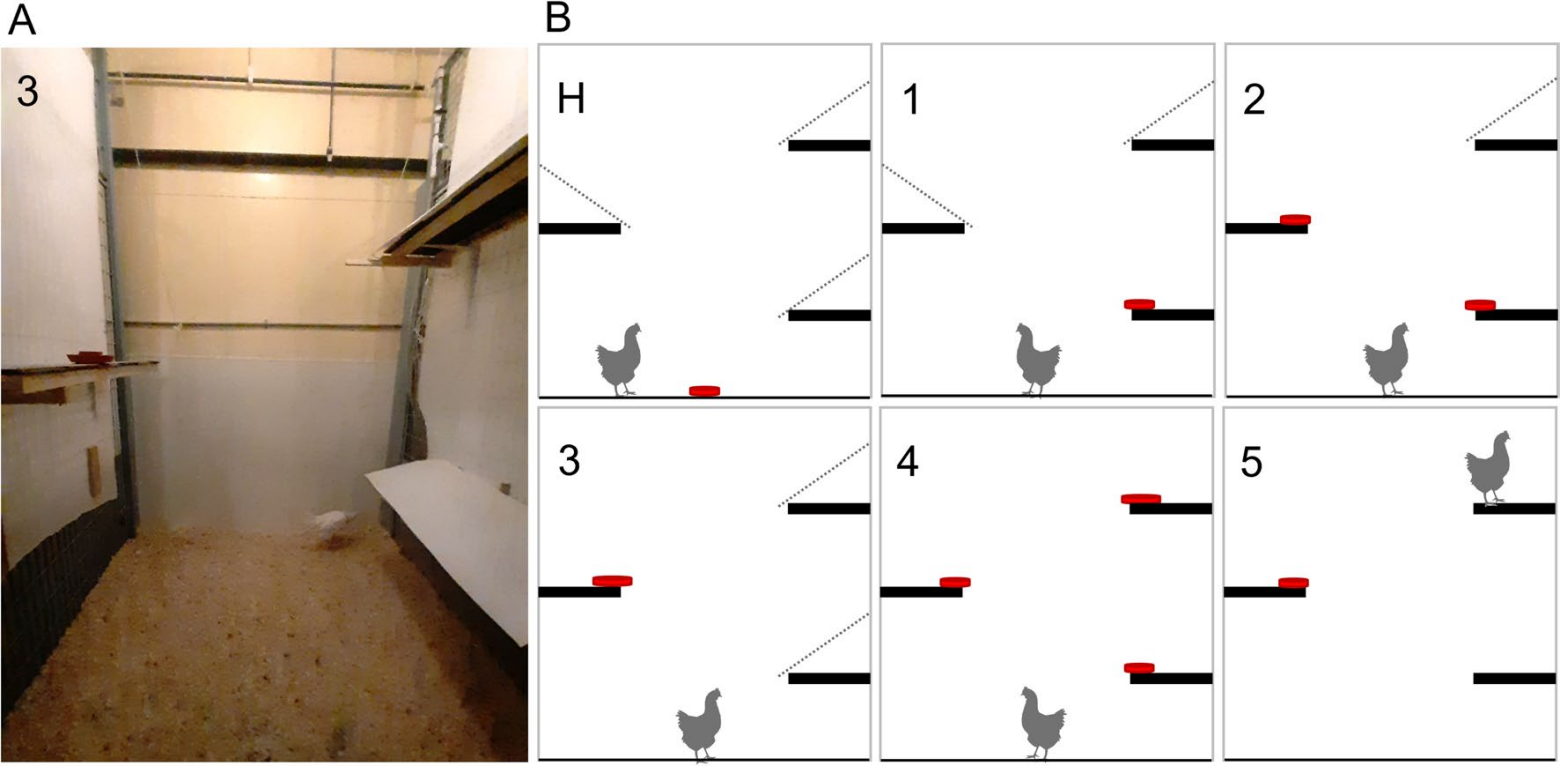
(**A**) A photo of a pullet during task 3 of the vertical navigation test. (**B**) Test pen of the vertical navigation test. Platforms were at 60 cm, 120 cm, and 180 cm height. During habituation (H), platforms were made inaccessible with a white plastic over (dashed line) and reward dish (red, mealworms and sweet corn) placed on the floor. For tasks 1 to 4, hens were placed on the floor, facing away from the reward dish. In task 5, hens were placed on the topmost platform, facing away from the reward. In tasks 2 and 4, success was defined as the hen reaching the highest reward with the lower dishes drawing the attention upwards but holding very little food. Schematics were created using Microsoft PowerPoint for Microsoft 365 MSO version 2304.

On the first day of week 15, 20 naïve pullets from each housing treatment and strain combination (160 pullets × 4 flocks) were moved from their rearing housing into floor pens furnished with an elevated perch (at 45 cm) and an elevated platform (at 70 cm) but no ramp. There were two testing pens with three platforms each (at 60 cm, 120 cm, 180 cm, Fig. 3). During week 15 of rearing, pullets were habituated to the testing pen and food reward dish. Due to time constraints a maximum number of 15 pullets per pen could be included in the test, therefore if more than 15 pullets met inclusion criteria (eating out of the reward dish on either day four, five, or both), surplus pullets were excluded randomly.

The following week (week 16 of rearing) included five days of testing in a modified version of a spatial test performed by Gunnarsson et al.^7^. Each day, all pullets were tested individually in a spatial task (see Fig. 3) with 5 min to complete it. In tasks 1–4, food rewards were placed on the elevated platforms and pullets were placed on the floor. For task 5, pullets were placed on the highest platform, with the reward being on the middle platform. Success was defined as both feet on the platform where the reward was located. The quality of each jump attempt was scored on a scale of 1–4 using the following criteria:

1. Jump attempt where the pullet did not reach the platform with her feet,
2. Pullet reached the platform with her feet but fell,
3. Pullet landed on the platform but had to balance by flapping her wings to stay on,
4. Pullet landed on the platform without the need for balancing^7^.

If no attempt was made within 5 min, the pullet was returned to her home pen without receiving a score. Is important to remark that when pullets received a score of 1 in task 5, it was not possible to discern the intention of reaching the reward on the platform and missing versus aiming for the litter area with a larger landing area.

### Ramp-choice test

**Figure 4:**
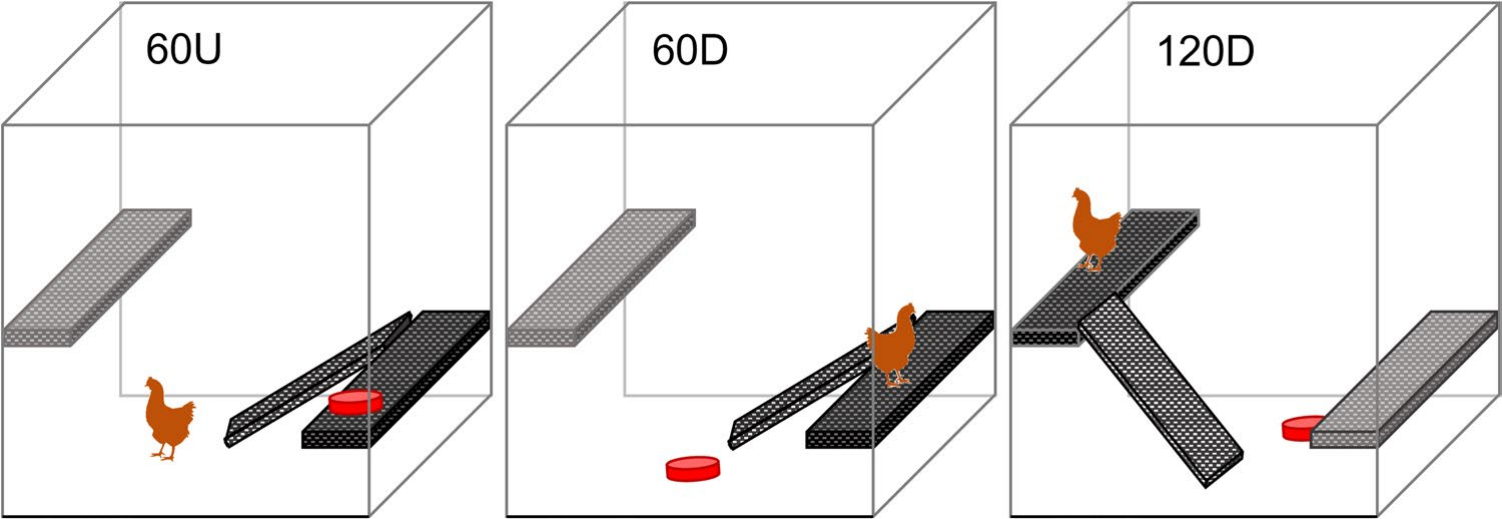
Test pen of the ramp-choice test. Ramps connected to the platforms at 60 cm and 120 cm at a 27° or 48° angle respectively. For tasks 60U, hens were placed on the floor, facing away from the reward dish (red). In tasks 60D and 120D, hens were placed on the platform, facing away from the reward. In each test, hens had the choice to navigate by aerial locomotion, by using the ramp or by a mix of both. Schematics were created using Microsoft PowerPoint for Microsoft 365 MSO version 2304.

In flock one and two, the same pullets used in the vertical navigation test were further tested in three vertical navigation tests including a ramp during week 17 (224 pullets). Ramps (black plastic grid on wooden frames) were added to the test pen, one leading from the ground to the first platform and a second one from the ground to the second platform (27° for the 60 cm platform, 48° for the 120 cm platform, Fig. 4). Pullets were given two minutes to complete the task and transition between the ground and the platform. For task ‘60U’, the red reward dish was placed on the platform at 60 cm elevation. In task ‘60D’, the reward dish was placed on the ground and the pullet on the platform at 60 cm elevation. For task ‘120D’, the reward dish was on the ground and the pullet was placed on the platform at 120 cm elevation. The outcome variables recorded were success, latency to succeed, and locomotion strategy of each attempted transition. Strategy was grouped into three categories:

- ‘Aerial’ vertical locomotion with both feet off the ground, can include wing use,
- ‘Ramp’ vertical locomotion along the ramp, can include wing use,
- ‘Mixed’ vertical locomotion involving both aerial and ramp.

### Data processing

#### Hurdle test

Six dependent variables were used to describe chick performance in the hurdle test: success (binary, yes = 1, no = 0), strategy (ramp vs jump), latency to walk, vocalisation, and persistence. The persistence to cross (number of crossing attempts made) was analysed for difficulties 1, 3, and 5 (884 observations) to minimize the risk of overestimating crossing attempts, as chicks occasionally appeared to ‘accidentally’ walk onto the ramp.

#### Vertical navigation test

Three dependent variables were used to describe pullet performance in the vertical navigation test: success, jumping quality, and strategy. An independent variable called ‘direction’ described locomotion required to complete a task as either upwards (tasks 1–4) or downwards (task 5). To analyse the quality of vertical navigation performed, ‘jumping quality’ scores were averaged over all five tasks. A total of 374 pullets attempted at least one jump.

#### Ramp-choice test

Three dependent variables were used to assess the performance in the ramp-choice tasks: success, latency to succeed, and strategy. Strategy was recorded for all attempted transitions (453 observations) as a categorical variable with three levels: ‘aerial’, ‘mixed’, or ‘ramp’.

### Statistical analyses

Statistical analyses were done in R and R Studio version 3.5.2. Dependent variables in the hurdle test were analysed by fitting generalized-/linear mixed effect models (G/LMM) with brooding compartment nested in housing style nested in flock as random effect. Models were validated visually for homoscedasticity and normality of residuals and random effect distribution. Persistence was analysed as the total amount of crossing attempts where success was used as one of the independent variables together with housing, strain, and all interactions in a GLMM.

Jumping quality in the vertical navigation test was analysed by fitting a LMM with housing, strain, and their interaction as fixed effects and home pen nested in flock as a random effect. Probability of success was analysed with a GLMM with housing, strain, direction (up: 1836, vs down: 459 observations), and their interactions as fixed effects and bird ID nested in flock (to account for repeated measures), and task (to account for repeated measure within “up” direction) as random effects.

Success and latency to succeed in the ramp-choice test were analysed by fitting a G/LMM with housing, strain, and their interaction as fixed effects. Bird ID nested in pen nested in flock were treated a random effect to account for repeated measures within the same individual and treatment group. Significance level threshold was set at an α of 5%. Main effects were assessed with a Wald Chi-square (χ^2^) test where effects that significantly contribute to the model fit would have a *p* value < 0.05. Post-hoc analysis of such factors was either done by applying a Tukey test with Bonferroni correction, or by calculating Odds ratios (OR). When interpreting OR, groups were considered different when the range of the 95% confidence interval (CI) did not include 1^40^.

A combination of non-parametric statistics and descriptive statistics were used to analyse locomotion strategy in the ramp-choice test. For each task, a Cochran–Mantel–Haenszel test was used to assess the dependency of strategy on housing and strain. If *p* < 0.05, strategy was assumed to be dependent on the combination of housing and strain. If *p* > 0.05, strategy was assumed to be independent from the interaction of housing and strain and the contribution of housing and strain were further assessed separately by calculating contingency tables. The contribution of a housing or strain group to strategy was assessed by comparing proportional chi-square values and visual representation of the data.

## Results

### Hurdle test

**Figure 5:**
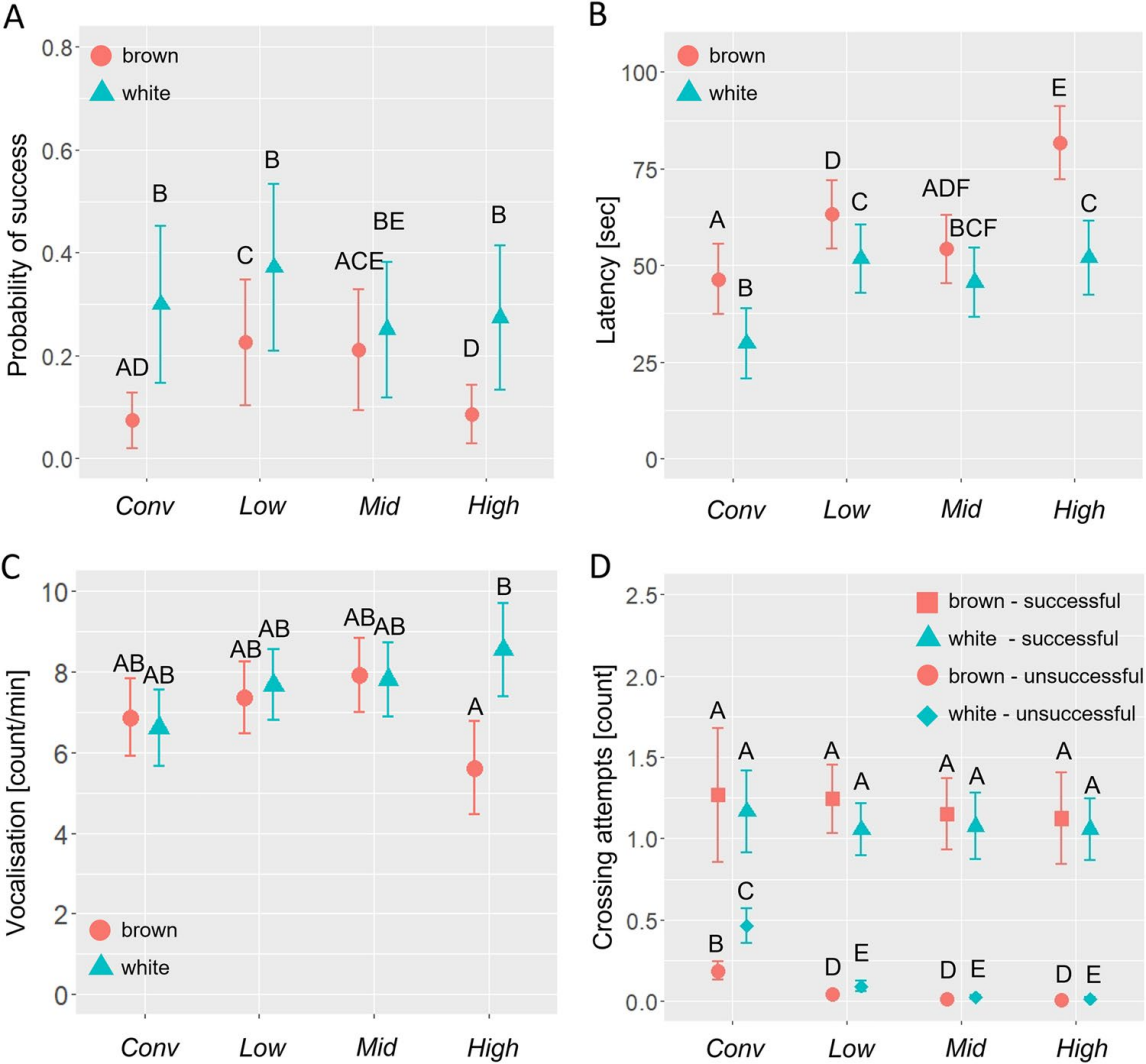
The probability of successful hurdle crossing (**A**) and measures of motivation for social re-instatement (**B**–**D**). (**A**) Estimated probability of success, (**B**) latency in seconds to start walking and (**C**) frequency of vocalisation bouts for each treatment group. Brown chicks are in circles (red) and white chicks in triangles (blue). (**D**) Persistence; the number of crossing attempts per test depending on success. Successful brown chicks are in squares (red), unsuccessful browns in circles (red), successful whites in triangles (blue), and unsuccessful whites in diamonds (blue). The rearing treatments are on the X-axis are in order of increasing spatial complexity; conventional cages [*Conv*], aviary with low [*Low*], intermediate [*Mid*], or high [*High*] complexity. The Y-axis reflects the estimated mean ± standard error. Different letters indicate a statistical difference according to the post-hoc analysis. Graphs were created using R and R Studio version 3.5.2.

*Probability of success* (raw values in Supplementary Table 2; model outputs in Table 1) was affected by housing (χ^2^ = 18.06, df = 3, *p* = 0.0004, Fig. 5A), strain (χ^2^ = 29.28, df = 1, *p* < 0.0001, Fig. 5A), and difficulty (χ^2^ = 150.12, df = 4, *p* < 0.0001) with no interactive effect of strain by difficulty (χ^2^ = 3.57, df = 4, *p* = 0.47), or housing by difficulty (χ^2^ = 15.96, df = 12, *p* = 0.19). Probability of success decreased with increasing difficulty up to difficulty level 3. Brown chicks had a lower estimated probability of success than white chicks (*p* < 0.0001). Chicks from *Low* were more likely to succeed than those from *Mid* (*p* = 0.02) or *High* (*p* = 0.0009), while *Conv* chicks varied widely but were not statistically different from any of the aviary chicks (95%-confidence interval of the estimated probability *Conv*: 0.001–0.67, *Low*: 0.26–0.39, *Mid*: 0.15–0.28, *High*: 0.13–0.25).

**Table 1:** Model output for the main outcome variables in the hurdle jumping test. *P* values based on the Wald chi-square test for fixed effects. Estimated means ± SEM for main effects.

	Success [probability]	Strategy [prob. of crossing by ramp]	Latency [seconds]	Vocalisation [rpm]	Persistence [# of attempts made]
Model	GLMM binary	GLMM binary	LMM	LMM	GLMM Poisson
# data points	1470	47	1470	1470	884
Housing	***p* = *0.0004***	*p* = *0.72*	*p* < *0.0001*	*p* = *0.5*	*p* = *0.001*
*Conv*	0.05 ± 0.09_ab_	0	38.12 ± 8.7	6.74 ± 0.84	0.23 ± 0.07
*Low*	0.32 ± 0.03_a_	0	57.44 ± 8.45	7.52 ± 0.77	0.13 ± 0.03
*Mid*	0.2 ± 0.03_b_	0.3 ± 0.13	49.9 ± 8.44	7.87 ± 0.77	0.05 ± 0.02
*High*	0.18 ± 0.03_b_	0.09 ± 0.09	66.79 ± 9.3	7.08 ± 1.11	0.02 ± 0.02
Strain	***p* < *0.0001***	*p* = *0.75*	*p* = *0.004*	*p* = *0.002*	*p* = *0.72*
Brown	0.12 ± 0.06_a_	0 ± 0.07	63.49 ± 8.28	6.59 ± 0.71	0.06 ± 0.02
White	0.29 ± 0.03_b_	0 ± 0.05	46.88 ± 8.28	7.81 ± 0.71	0.11 ± 0.02
Housing × strain	*p* = *0.18*	–	***p* = *0.003***	***p* = *0.004***	*p* = *0.98*
Additional significant explanatory variable					
Success: difficulty (*p* < *0.0001*)					
Persistence: housing × success (***p* < *0.0001***)					
Persistence: strain × success (***p* = *0.01***)					

Bolded *p* values indicate significant interaction effects or main effects not driven by interactions.

a-b: different letters indicate a statistical difference

“rmp” = rate per minute.

“prob.” = probability.

#### Strategy

Crossing strategy (jumping vs ramp) was not affected by either housing (χ^2^ = 1.3, df = 3, *p* = 0.72) or strain (χ^2^ = 0.1, df = 1, *p* = 0.75, Table 1). A total of 154 chicks (58 browns, 96 whites) out of 588 succeeded in the ramp tests, of those, 27 used the ramp, and the remaining jumped.

*Motivation* (model outputs in Table 1; estimated means in Fig. 5B–D). Latency to walk after placement in the test was affected by an interaction of housing and strain (χ^2^ = 13.67, df = 3, *p* = 0.003, Fig. 5B, Table 1). *Conv* chicks in both strains had a shorter latency to walk than their counterparts in *Low* (B: t = − 2.83, *p* = 0.04, W: t = − 3.71, *p* = 0.006) or *High* (B: t = − 35.16, *p* = 0.0001, W: t = − 22.18, *p* = 0.01). *High* brown chicks had longer latencies than chicks in all other treatment groups. Browns had longer latencies than whites in *Conv* (t = − 16.65, *p* = 0.004), *Low* (t = − 11.50, *p* = 0.02), and *High* (t = − 29.65, *p* < 0.0001). Vocalisation frequency was affected by an interaction of strain and housing (χ^2^ = 13.31, df = 3, *p* = 0.004, Fig. 5C) with white *High* chicks vocalising more frequently than *High* browns (z = 4.8, *p* < 0.001). Persistence (number of crossing attempts made, Fig. 5D) was affected by interactive effects of housing by success (χ^2^ = 38.87, df = 3, *p* < 0.001) and strain by success (χ^2^ = 6.43, df = 1, *p* = 0.01). Successful chicks performed more crossing attempts than unsuccessful chicks in all treatment groups. Unsuccessful chicks from *Conv* performed more crossing attempts than unsuccessful chicks from all other housing groups, regardless of strain [*Low* (z = 4.4, *p* < 0.001), *Mid* (z = 4.9, *p* < 0.001) and *High* (z = 4.7, *p* < 0.001)]. Unsuccessful brown chicks performed fewer jumping attempts than unsuccessful white chicks in all housing groups (z = − 2.6, *p* = 0.01). There were no differences in crossing attempts of successful chicks due to either housing or strain.

### Vertical navigation test

**Figure 6:**
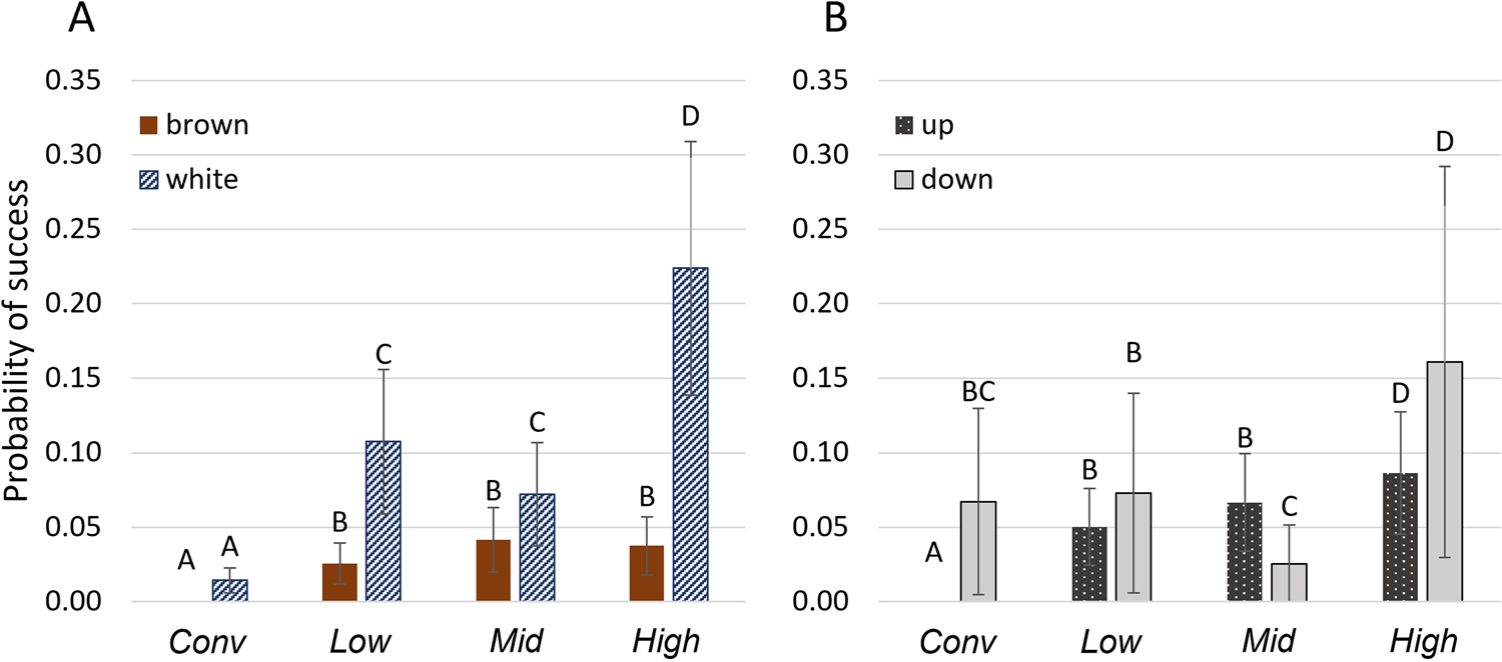
Probability of success in the vertical navigation test (all 5 tasks combined) according to housing system. The probability was affected by (**A**) a housing by strain interaction where browns are in brown and whites in blue (patterned) and (**B**) a housing by jumping direction interaction with upwards tasks (tasks 1–4) in black (patterned) and the downward task (task 5) in grey. Different letters indicate a statistical difference according to the post-hoc analysis. Graphs were created using Microsoft Excel for Microsoft 365 MSO version 2304.

*Success* in the vertical navigation task (raw data) is detailed in Supplementary Table 2 with model outputs for success across all five tasks given in Table 2. Probability of success was affected by a housing by strain interaction (χ^2^ = 8.64, df = 3, *p* = 0.03, Fig. 6A) and a housing by direction interaction (χ^2^ = 17.34, df = 3, *p* = 0.0006, Fig. 6B). *Low* white pullets had 3.02 times higher odds of reaching the reward than browns (95% confidence interval [CI] = 1.82, 5.19). In *Mid* whites had 1.68 times higher odds of success than browns (CI = 1.03, 2.79) and in *High*, whites had 4.26 times higher odds of success than browns (CI = 2.74, 6.81). Aviary-reared birds had higher odds of success than *Conv* pullets, regardless of strain. In whites, *Low* pullets had 4.54 times (CI = 2.57, 8.52), *Mid* had 3.49 times (CI = 1.95, 6.6), and *High* had 8.37 times (CI = 4.85, 15.41) higher odds of success than *Conv* pullets. In whites, *High* pullets had 2.4 times higher odds of success than *Mid* pullets (CI = 1.62, 3.57) and 1.84 times higher odds than *Low* (CI = 1.03, 2.7).

**Table 2:** Model output for outcome variables in the vertical navigation test. *P* values based on the Wald chi-square test for fixed effects. Estimates ± standard errors for main effects.

	Success [probability]	Quality [^1^mean score]	Strategy [prob. of multi]
Model	GLMM binary	LMM	GLMM binary
# data points	2295	374	74
Housing	*p* = *0.0004*	***p* < *0.0001***	*p* = *0.07*
*Conv*	0 ± 0.0003	1.69 ± 0.26^a^	0 ± 0.0005
*Low*	0.05 ± 0.03	2.56 ± 0.26^b^	0.25 ± 0.11
*Mid*	0.06 ± 0.03	2.49 ± 0.25^b^	0.16 ± 0.09
*High*	0.1 ± 0.04	2.78 ± 0.25^b^	0.56 ± 0.12
Strain	*p* < *0.0001*	*p* = *0.5*	*p* = *0.74*
Brown	0.001 ± 0.008	2.35 ± 0.24	0.32
White	0.08 ± 0.03	2.44 ± 0.24	0.27
Direction	*p* = *0.73*	–	–
UP	0.007 ± 0.02	–	–
Down	0.07 ± 0.06	–	–
Housing × strain	***p* = *0.03***	*p* = *0.08*	–
Housing × direction	***p* = *0.0006***	–	–
Strain × direction	*p* = *1.4*	–	–
Housing × strain × direction	*p* = *0.73*	–	–

Bolded *p* values indicate significant interaction effects or main effects not driven by interactions.

a-b: different letters indicate a statistical difference.

^1^scores: ‘1’: platform not reached, ‘2’: platform reached but fell, ‘3’: success with balancing, ‘4’: perfect landing.

“prob.” = probability.

“multi” = multiple jumps used to reach highest reward.

*P* values based on the Wald chi-square test for fixed effects. Estimates ± standard errors for main effects.

When navigating upwards (tasks 1–4; Fig. 6B), *Low* had 9.02 times higher odds (CI = 4.5, 20.67), *Mid* had 9.85 times higher odds (CI = 4.94, 22.52), and *High* had 14.13 times higher odds (CI = 7.18, 32.08) of reaching the reward than *Conv*. *High* had 1.57 times higher odds of success navigating upwards than *Low* (CI = 1.1, 2.25) and 1.44 times higher odds than *Mid* (CI = 1.01, 2.04). Jumping downwards (task 5), *High* pullets had higher odds of reaching the reward than *Mid* pullets (OR = 5.07, CI = 2.22, 13.13), 1.99 times higher odds than *Low* (CI = 1.02, 3.99) and 2.21 times higher odds than *Conv* pullets (CI = 1.11, 4.59). *Low* pullets had 2.55 times higher odds of success moving downwards than *Mid* pullets (CI = 1.04, 6.9). Pullets raised in *Conv*, had 7.87 times higher odds of success moving down compared to upwards (CI = 3.28, 20.42), while *Mid* pullets had 2.72 times higher odds of success moving upwards than down (CI = 1.3, 6.67).

*Jumping quality* was affected by housing (χ^2^ = 42.75, df = 3, *p* < 0.0001) but not strain (χ^2^ = 0.45, df = 1, *p* = 0.5) or their interaction (χ^2^ = 6.74, df = 3, *p* = 0.08, Table 2). Pullets raised in *Conv* had a lower mean jumping score than any of the aviary reared pullets. Mean quality scores might have been inflated by task 5, where scores of 1 were highly prevalent (52.72% of pullets; B: 43.5%, W: 61.4%) even if the pullet’s intent was unclear (aim for platform with food and miss or aim for litter floor to escape).

### Ramp-choice test

*Success* was affected by housing (χ^2^ = 35.97, df = 3, *p* < 0.0001) but not by strain (χ^2^ = 2.8, df = 1, *p* = 0.09) or their interaction (χ^2^ = 6.6, df = 3, *p* = 0.09). Across all three tasks and both strains, *Conv* pullets were successful 37.3% of the time, *Low* 69.2%, *Mid* 66.7% and *High* 75.6% of the time. All aviary pullets had higher odds of success compared to *Conv* pullets (*p* < 0.001).

*Latency to succeed* was affected by housing (χ^2^ = 12.73, df = 3, *p* = 0.005) and strain (χ^2^ = 3.96, df = 1, *p* = 0.046) but not their interaction (χ^2^ = 3, df = 3, *p* = 0.4). Brown pullets took longer to succeed (18.3 ± 1.12 s) than white pullets (14.2 ± 1.12 s). Pullets from *High* (12.5 ± 1.14 s) were faster to succeed than pullets from *Conv* (24.9 ± 1.19 s, *p* = 0.03).

#### Strategy

**Figure 7:**
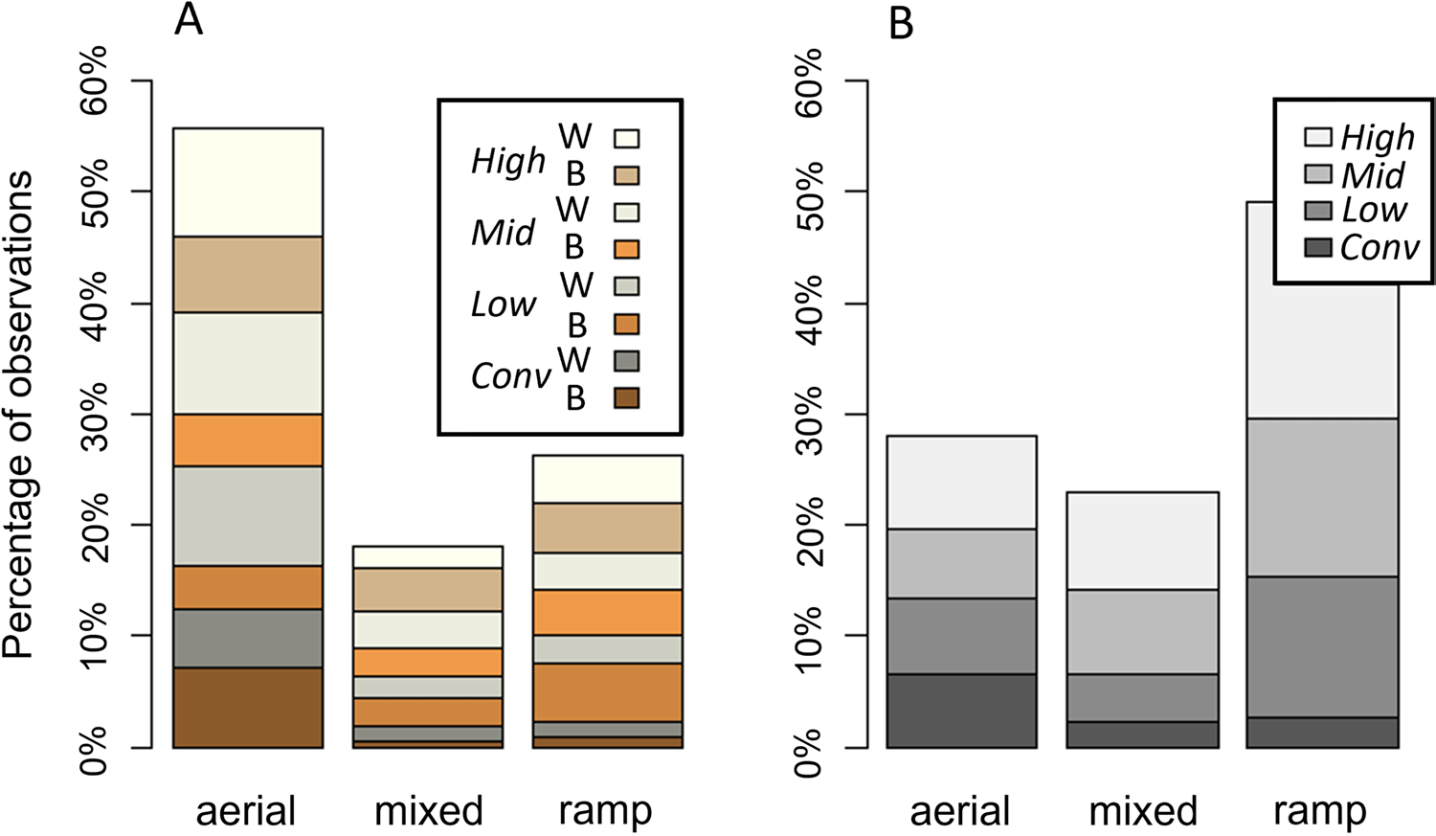
Locomotion strategy used in the ramp-choice test as percentage of observations. The different strategies are aerial locomotion, a combination of aerial and ramp use (mixed), or exclusive use of the ramp. The different bands represent the proportion of pullets from each treatment group with housing complexity increasing from the bottom bands to the top bands (dark to bright colours). Data includes all attempted transitions regardless of success. (**A**) Task 60U (up 60 cm): 55.68% of pullets used aerial locomotion, 18% used a mix of aerial and the ramp, and 26.32% exclusively used the ramp. Strategy was dependent on housing and strain (Cochran–Mantel–Haenszel [CMH] M^2^ = 7.8, *p* = 0.02), brown birds in tan-scale, whites in grey-scale). (**B**) Task 60D (down 60 cm): 27.97% of pullets used aerial locomotion, 22.99% used a mix, and 49.04% used the ramp only. Strategy was independent on housing but not strain (housing × strain: CMH M^2^ = 4.1, *p* = 0.1; housing: chi-square [χ^2^] contribution to aerial: *Conv* χ^2^ = 8.8, *Low* χ^2^ = 0.03, *Mid* χ^2^ = 1, *High* χ^2^ = 0.9, ramp: *Conv* χ^2^ = 0.1, *Low* χ^2^ = 0.7, *Mid* χ^2^ = 0.6, *High* χ^2^ = 0.04, mixed: *Conv* χ^2^ = 4, *Low* χ^2^ = 0.2, *Mid* χ^2^ = 0.04, *High* χ^2^ = 0.3). Graphs were created using R and R Studio version 3.5.2.

A Contingency table describing the dependency of strategy and housing by task is detailed in Table 3 while there appeared to be no strong associations between strategy, task, and strain. Each task was assessed individually. In task 60U, the overall strategy of choice was aerial locomotion followed by ramp only, and mixed locomotion was used least (Fig. 7A). Strategy was dependent on housing and strain (Cochran–Mantel–Haenszel [CMH] M^2^ = 7.8, df = 2, *p* = 0.02). The preference for aerial locomotion appeared most pronounced in *Conv* pullets where any ramp use (mixed or ramp) was rare. The preference was least distinct in brown aviary-reared pullets and *Low* browns even had a preference of ramp use over aerial locomotion. In task 60D, ramp use was most prevalent overall (Fig. 7B). The strategy chosen was independent from housing and strain (CMH M^2^ = 4.1, df = 2, *p* = 0.1). Assessing the contingency tables for housing and strain contribution (see Table 3 for housing contribution), *Conv*-pullets choosing aerial locomotion had the largest proportional chi-square contribution (*χ*^2^ = 8.83, Table 3). This contribution of housing on the strategy can be explained by *Conv* pullets being the only group that preferred aerial locomotion over the other forms in this task (Fig. 7B). In task 120D, aerial locomotion was again preferred (53.62% of attempted transitions), sole ramp use next (31.52% of attempted transitions), and mixed strategy least (14.86% of attempted transitions). Strategy was independent of housing and strain (CMH M^2^ = 1, df = 2, *p* = 0.6) and there was no indication for one treatment group contributing seemingly more than another in the contingency tables.

**Table 3:**
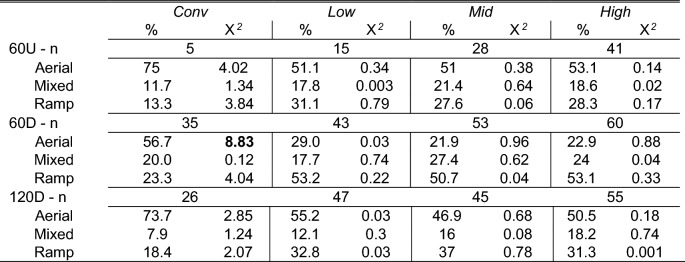
Contingency table strategy × housing. Locomotion strategy used in the ramp-choice test depending on rearing style. Per housing style, the percentage of pullets using a given strategy is given as well as the proportional chi-square (2) contribution. The indicated n refers to the number of birds that attempted a transition in each task. The total numbers of birds tested were 50 from *Conv*, 58 from *Low*, 55 from *Mid*, and 60 from *High*.

Bolded χ^2^ values are thought to contribute proportionally more to the data distribution than other groups.

Locomotion strategy used in the ramp-choice test depending on rearing style. Per housing style, the percentage of pullets using a given strategy is given as well as the proportional chi-square (χ^2^) contribution. The indicated n refers to the number of birds that attempted a transition in each task. The total numbers of birds tested were 50 from *Conv*, 58 from *Low*, 55 from *Mid*, and 60 from *High*.

## Discussion

To the authors’ knowledge, this is the first study to assess 3D spatial skills in laying hen pullets raised with different degrees of environmental complexity in commercial aviary systems while allowing for a direct comparison of brown and white feathered strains.

We developed the hurdle test to assess the vertical spatial skills of chicks at the end of the brooding phase. The vertical navigation test conducted toward the end of rearing was intended to assess the pullets’ physical and navigation skills in a more difficult and age-appropriate task. Following the vertical navigation test, the ramp-choice test assessed success and locomotory vertical locomotion strategy when given a choice by the provision of a ramp. These latter tests were conducted at an age that is close to when pullets would be transferred to complex layer aviaries in commercial systems and would need these skills to adapt well ^8^. We hypothesized that chicks from more complex brooding compartments would be more successful (*High* > *Mid* > *Low* > *Conv*) and that white birds would perform better than brown. Contrary to our predictions, chicks from *Low* were most likely to succeed in the hurdle test; however, in brown chicks from *High*, motivation measures (latency to walk and vocalization frequency) pointed towards lower motivation for social reinstatement rather than a lack in physical or spatial ability. In the vertical navigation test, pullets from rearing aviaries were more likely to succeed and performed jumps of better quality than those raised in *Conv*. Additionally, white *High* pullets were more likely to succeed than white *Mid* and *Low* pullets. The direction of the strain differences was as predicted, as white chicks and pullets performed better than browns in all tests. Contrary to our expectations, we found no strain differences in the success or preferred locomotion strategy in the ramp-choice test. Most birds preferred aerial locomotion when moving upwards. As expected, only aviary-reared pullets preferred to use the ramp (27° decline) when moving downwards from 60 cm. Most pullets preferred aerial locomotion when placed at 120 cm with a ramp at a 48° decline. For ease of conceptional comparison, we ranked the aviaries in terms of complexity as Low, Mid, and High. However, it should be acknowledged that because these were commercial models, some differences were qualitative rather than quantitative and level of complexity was not always linear. During brooding, for example, the *Mid* style aviary required birds to negotiate a greater range of vertical space while the horizontal space was significantly smaller compared to *High*. Additionally, during the open phase, *Low* and *Mid* were quite similar to one another and both considerably different from *High*.

### Gene by environment interaction

White chicks were more likely to succeed in the hurdle jumping test than brown chicks regardless of housing, and white pullets were more likely to succeed in the vertical navigation task than browns when they were reared in aviaries. Aviary-reared pullets were more successful than *Conv* birds with no strain effects in the ramp-choice test. In the vertical navigation test, between aviary effects were only detected in white pullets but not in browns, with white pullets from rearing environments with high complexity (*High*) being more successful in the vertical navigation test than those from less complex rearing aviaries (*Mid* and *Low*). Versions of the vertical navigation test have been used in previous studies to test for the effects of early access to spatial structures on spatial abilities. Access to perches before eight weeks of age improved the success and speed at which 16-week-old pullets moved between elevated platforms^7^. Early access to perches, a platform, and a ramp did not, however, improve the performance in a vertical jump test of two or four-week-old chicks, though there was an improvement with age^41^. However, the tests in that study were done in brown feathered pullets only.

Strain differences^42,43^ and the interaction of aviary style by strain^28^ have been reported in the literature regarding locomotory behaviours performed in aviaries. When observed on commercial farms, white pullets locomoted more and performed more aerial locomotion regardless of aviary style, and white pullets in the most complex aviary performed most vertical transitions^28^. The present study was part of a larger project where birds were observed in smaller versions of commercial rearing aviaries, and both white and brown chicks locomoted most in complex brooding compartments (*High*)^35,44^. However, only white pullets still performed more locomotory behaviours in *High* and *Mid* than *Low* after the brooding compartments were opened, further suggesting that rearing aviary design might affect white feathered birds more or for longer than browns.

### Upwards vs downwards

Focussing on downwards movements only, *High* pullets, which were more successful than *Mid* and *Low* pullets, were the only aviary group different from *Conv* pullets, while *Low* pullets surprisingly did better than *Mid* pullets. *Conv* pullets were more likely to succeed when asked to move down than upwards, and the opposite was true for *Mid* pullets. The poor performance of *Mid* pullets in downwards movements could either be an artifact of the test (type I error) or an effect specific to the experimental versions of the rearing aviaries and, therefore, not applicable to *Mid* systems on the commercial scale that are much larger in scale. In previous studies, downwards movements were reported to be more difficult for laying hens than upwards movements, shown by a refusal to transition or transitions resulting in failed landings^33,34^. Laying hens appear to have a limited control over their downwards flight trajectory and velocity based on their wing and whole-body kinematics in a jump tower experiment^45^. In commercial aviaries, failed landings after downwards transitions were more often observed when landing on perches than when landing on litter^6^. Strain differences have also been reported to be direction specific. The force (energy) experienced at the keel at take-off was lower for whites than browns completing downwards movements and greater when distance increased in upwards movements^46^. We did not replicate a strain difference in based on direction of the jump, though this could be due to an underrepresentation of downward tasks in our experiment.

### Motivation

Tests performed on chicks often use social reinstatement as a reward over food ^41^, as chicks reportedly prefer social rewards over food rewards^39^. Also, social isolation tends to interfere with chick performance in cognitive tasks^47^. The hurdle task was designed to test navigational skills in chicks, but our results suggest a difference in motivation for social reinstatement not only between brown and white feathered chicks but also between housing systems (most motivated: *Conv* whites, least motivated: *High* browns). One possible explanation could be that the different housing systems led to differences in motivation for social reinstatement. In *High*, chicks were able to become accustomed to larger inter-bird distances due to the larger total floor space, whereas the confinement in the other housing systems during the brooding period (first few weeks) did not allow for that. Lower motivation in brown layers for social cues and food rewards has been reported previously^27,48^, though brown chicks were seen to vocalize more in social isolation tests^49^. While browns might be more anxious than whites when isolated, they may be less motivated to reduce inter-bird distance once conspecifics are in sight. Alternatively, their high levels of anxiety due to social isolation could have affected their confidence in performing the task. In the ramp-choice test, browns were slower to succeed than white pullets, indicating either a lower motivation to access the food-reward or an overall slower pace in browns. Performance differences in white pullets from the different housing systems in both of our tests were most likely due to differences in skills, while the overall low motivation in browns makes it difficult to differentiate between *cannot* and *will not* in the hurdle test. However, it seems more likely that differences in performance were based on capability, as pullets were excluded from testing if they did not appear interested in the food reward. Compared to the study by Gunnarsson and colleagues^7^ the low motivation in browns and the overall low participation rate could be explained by our decision not to food-deprive pullets before testing. Food-depriving pullets for several hours before each task (15 h in Gunnarsson et al.^7^) would increase the motivation for a food reward. Additionally, our test was more difficult with more levels, more tasks, and platforms at higher heights (60 cm, 120 cm, 180 cm vs. 40 cm, 80 cm, 160 cm). However, the low or non-existent participation of pullets raised in conventional cages likely indicates their inability to perform the task, either physically or cognitively.

### Physical vs cognitive abilities

Locomotion within 3D space requires physical skills in the form of muscle mass and coordination as well as cognitive spatial skills^7^. It has been previously suggested that in the right environment, vertical locomotion skills might come easily to laying hens, as night-time roosting is an anti-predator behaviour which is usually innate^50^. Hence, the early life environment should support cognitive understanding of the concept of multiple levels^9^. Gunnarsson and colleagues^7^ supported this sentiment when they concluded that rearing with perches affected the development of cognitive spatial skills rather than physical skills. Contrary to this, in our hurdle test, chicks from *Conv* were most persistent but least successful in crossing the hurdle, indicating that they cognitively understood the task but were not physically able to perform it. Furthermore, in another study, rearing with perches, ramps and platforms to enhance physical skills did not improve the performance of two or four-week-old chicks in a vertical jump test^41^.

In the vertical navigation test, only one brown pullet from a conventional cage attempted a jump. In contrast, a handful of whites from *Conv* attempted, and some even succeeded in reaching a platform. Hence, at least in white pullets, rearing without elevated platforms during early life did not eliminate their physical capabilities to navigate them. All pullets were housed with elevated platforms in the floor pens, though brown *Conv* pullets did not appear to use them (anecdotal observations). The success of these few white *Conv* pullets agrees with the theory that once laying hens learn of the existence of a third dimension, they possess the physical capabilities to navigate it ^7,9^ However, *Conv*-reared birds lack flight muscle weight as the opportunities for their development was not on par with aviary-reared birds^36,51^.

The importance of a 3D understanding was further demonstrated in the ramp-choice test and the ramp options during the hurdle test. Theoretically, ramp use does not require any flight skills and all birds should have been able to use it. However, chicks preferred to jump rather than use the ramp during the hurdle test. Chicks had no prior experience with ramps, and they might not have comprehended their function. In both strains, pullets with ramp experience used ramps at a 27° angle to move down, while an angle of 48° discouraged ramp use. These findings support previous suggestions of a maximum ramp incline of 40° to promote easy negotiation of complex housing systems^52^. In accordance with our results, rearing with ramps improved speed and success when using ramps^53^ and even improved long-term spatial distribution of birds^23^. In the present hurdle test, almost two-thirds of those who used the novel ramp were white chicks, while numbers were close to equal between strains once the ramp was no longer new. In the vertical navigation test, white pullets raised in *High* performed better than white pullets raised in *Low* and *Mid*, while no aviary differences were observed in brown pullets. Whether that was due to a cap on physical skills or aviary designs not catering to the needs of brown pullets remains to be seen. To disentangle *cannot* and *will not*, a test would need to be developed with an incentive that motivates brown and white strains equally while controlling for the effect of social isolation. Overall, white birds appear to be more behaviourally flexible.

## Conclusion

Genetic strain predicted performance in both spatial tests (whites > browns) while impacts of type of rearing housing were primarily reflected in the difference between aviary-reared and conventional cage-reared birds. Aviary design effects appeared to be strain-specific; white pullets raised with high complexity more successfully navigated 3D space than those raised in moderate or low complexity (*High* > *Mid*, *Low*), which was not seen in browns. A lower motivation for test participation were found in brown compared to white chicks, with potential ramifications for their differences in test performance as pullets. Our findings support a gene by environment interaction in the development of spatial skills in laying hens with white feathered pullets but not browns improving their vertical navigation skills in line with increasing degree of spatial complexity.

## Supplementary Information

Supplementary Information.

## Data Availability

The data supporting the presented findings and conclusions are openly available on the Canadian Dataverse Repository Borealis under https://doi.org/10.5683/SP3/VXMBJV.
