# Supplementary material for: The development of laying hen locomotion in 3D space is affected by early environmental complexity and genetic strain
Source: Sci Rep. 2023 Jun 21;13:10084. doi: 10.1038/s41598-023-35956-1 (PMC10284819; doi:10.1038/s41598-023-35956-1)
Supplement: Supplementary file 1 — Supplementary Information. [file 41598_2023_35956_MOESM1_ESM.pdf]

Methods

Housing

The different housing treatments consisted of three styles of rearing aviaries incrementally increasing in environmental complexity (*Low*, *Mid*, and *High*) and conventional pullet cages (*Conv*). The rearing aviary designs had increasing spatial complexity and differed most during the brooding phase (first 6 weeks) where chicks are typically confined to the brooding compartment for safety and management (Figure 1).

Each housing system housed a different number of birds according to the manufacturer's recommendations and in line with the Canadian Code of Practice [1]. The least complex rearing aviary (*Low*) had three brooding compartments that held 115 chicks each. Each compartment was furnished with two elevated perches (*Low* n=345per strain and flock). The intermediate aviary (*Mid*) also had three brooding compartments; they each held 144 chicks and offered three elevated perches as well as an elevated platform (*Mid* n=432 per strain and flock). The most complex aviary (*High*) had one brooding compartment that held 600 chicks and spanned the whole length of the barn. It offered 6 elevated perches and an elevated platform (*High* n=600 birds per strain and flock). Conventional cages (*Conv*) were barren cages that held 30 chicks during the brooding phase and 15 until the end of rearing, with no change in environmental complexity (*Conv* n=120 per strain and flock). While the sample size (n=8/ housing treatment) was determined by feasibility (time and infrastructure constrains), the number of individuals (N) used per test was justified by calculating the degrees of freedom [2,3] needed to analyse population variation (Supplementary Tables 1, 3, and 5).

Hurdle test

The hurdle test was conducted in a testing arena in a separate room. The testing arena consisted of two sections, divided by a hurdle (Figure 2). The bigger section held a group of three chicks as a social incentive for attracting the focal bird to cross the hurdle. Feed was strewn on the floor to keep the incentive chicks engaged in front of the hurdle, and a mesh barrier prevented them from jumping over the hurdle into the testing section. The testing section was smaller and offered no feed. The hurdles were made of white plastic grid that allowed chicks to see through to the other side but prevented them from passing through.

Each chick was tested in one of five possible set-ups only, with 294 chicks tested per set-up. See Supplementary Table 2 for a detailed description of the numbers of chicks tested by housing system. In the two set-ups that included a ramp (difficulties 2 and 4), ramps were at a 30- or 42-degree angle for difficulties two and four respectively. Chicks of this age have been seen to climb ramps of up to 40-degrees incline without much difficulty with increasing struggle at steeper angles [4]. Sample size for each difficulty level consisted of 12 chicks per aviary and strain combination and 6 of both strains of conventional cage chicks for flocks 2-4. In flock 1, only half as many chicks were tested in each group. This resulted in a total of 210 browns and 210 whites tested from each of the aviary styles and 105 of each strain from the conventional cages.

Supplementary Table 1. Power analysis for the hurdle jumping test based on the number of degrees of freedom (df) generated and used.

	df		df
**Total**		**Predictors**	
housing	4	housing	3
strain	2	strain	1
flocks	4	difficulty	4
N Flock 1: 6/ aviary/ strain, 3/ conv/ strain		housing x strain	3
N Flocks 2-4: 12/ aviary/ strain, 6/ conv/ strain		housing x difficulty	12
total df=	294	strain x difficulty	4
**Random**		predictor df =	31
housing	4	**Residual**	
strain	2	df tot.- df rand.-intercept-df pred.	
flock	4	residual df=	174
brooding compartments/ strain			
High: 1, *Mid*: 3, *Low*: 3, *Conv*: 4			
random df=	88		

Supplementary Table 2. Number of birds that succeeded in the hurdle test by difficulty (diff.) and vertical navigation test by task for each housing by strain combination, and percentage (%) of successful birds for each housing group. The number of birds that were tested in the given difficulty/ task is given by N.

Housing and strain	N/ diff.	Hurdle test 1	2	3	4	5	N/ task	Vertical navigation test 1	2	3	4	5
Conv	**42**	**19**	**8**	**7**	**5**	**2**	**108**	**8**	**9**	**3**	**3**	**83**
%		45.2	19.1	16.7	11.9	4.8		7.4	8.3	2.8	2.8	76.9
brown	21	4	4	3	2	0	50	0	1	0	0	39
white	21	15	4	4	3	2	58	8	8	3	3	44
Low	**84**	**47**	**37**	**23**	**19**	**15**	**116**	**19**	**35**	**25**	**30**	**76**
%		56.0	44.0	27.4	22.6	17.9		16.4	30.2	21.6	25.9	65.5
brown	42	21	14	11	8	4	58	5	8	8	9	29
white	42	26	23	12	11	11	58	14	27	17	21	47
Mid	**84**	**51**	**26**	**17**	**16**	**2**	**115**	**21**	**40**	**30**	**39**	**75**
%		60.7	31.0	20.2	19.0	2.4		18.3	34.8	26.1	33.9	65.2
brown	42	24	9	8	7	1	55	10	17	12	16	25
white	42	27	17	9	9	1	60	11	23	18	23	50
High	**84**	**40**	**33**	**9**	**10**	**6**	**120**	**35**	**50**	**29**	**41**	**84**
%		47.6	39.3	10.7	11.9	7.1		29.2	41.7	24.2	34.2	70.0
brown	42	12	9	2	5	2	60	11	14	6	12	34
white	42	28	24	7	5	4	60	24	36	23	29	50

Testing occurred over a period of four days, with chicks from each housing and strain combination tested in a balanced order on each day. Test difficulty was randomized in flock 1 but switched to a standardized increase over the testing period for the three following flocks to reduce the time spent on remodelling the arena in favour of testing more birds. Three conspecifics were used as a cue to motivate the chicks to cross the hurdle for social re-instatement. To start a test, the focal chick was placed in the testing section (centre and opposite the hurdle, see Figure 2), facing the hurdle. The observation started as soon as the chick was set down and continued

for 120 sec or until the chick succeeded in crossing the hurdle. Chicks were observed live by one of two observers, and behaviour coded with a behaviour coding program on digital tablets (Pocket Observer 3.2 by 2012 Noldus Information Technology on Samsung Galaxy Tab4 SM-T230NU). The observation period started when the chick was placed (both feet touched the floor) and behaviours measured were vocalisation bouts, latency to start walking, the number of jumping attempts made, crossing success, and strategy (ramp vs jumping). Inter-observer reliability was assessed by the two observers coding a total of 13 tests simultaneously. The number of birds tested by housing system and the success for each difficulty are detailed in Supplementary Table 2.

Vertical navigation test

The vertical navigation test was conducted in two test pens while the focal birds were homed in floor pens for the duration of this test. Every pullet was individually marked with numbered leg ring and numbered wing tag. All birds from a given housing treatment were randomly assigned to a pen with the experimenter blinded to the treatment. All pens were located in the same room and blinding required that the strains were kept on opposite sides of the room, to allow switching location of treatment groups with every flock. At the end of the room, there were two testing pens with three platforms each (at 60 cm, 120 cm, 180 cm, Figure 3). All platforms were made of black rubber grid fixed on metal frames.

Supplementary Table 3. Power analysis for the vertical navigation test based on the number of degrees of freedom (df) generated and used.

	df		df
**Total**		**Predictors**	
housing	4	housing	3
strain	2	strain	1
flocks	4	direction	1
N/ flock/ housing/ strain	10†	housing x strain	3
total df=	320	housing x direction	3
**Random**		strain x direction	1
housing	4	housing x strain x direction	3
strain	2	predictor df =	15
flock	4	**Residual**	
task	5	df tot.- df rand.-intercept-df pred.	
random df=	160	residual df=	144

† The expected number of pullets to qualify for testing, there ended up being more in every group except for *Conv* browns in flock 1.

Habituation. During the 1st week in the floor pens (week 15 of rearing), pullets were first introduced to the food reward, reward dish (red), and habituated to the test pens (Figure 3). Habituation week went as follows: Day one, feeders were removed from the home pens for 30-60 minutes (min) before each pen received a red dish with high value foods (sweet corn and live mealworms) mixed into their normal feed. On days two and three, feeders were removed from the pens for 30-60 min before pullets were placed into the test pens, in groups of five, for 20 min with two reward dishes on the floor, holding reward foods. Before returning feeders into the home pens, all pens received one more portion of the food reward in the reward dish on the floor of their home pens to further strengthen association. On days four and five, after removing the feeder for 30-60 min, pullets were placed individually into the test pen for 3 min and interaction with the reward dish was recorded (avoidance, inspection, eating). On these days, all pens received reward food in two reward dishes in their home pens twice, each time one dish was placed on the floor, the other on the elevated platform. This was done to introduce pullets to the option of feeding on the platform. At the end of day five, pullets that did not meet the inclusion

criteria, i.e., eating out of the reward dish on either day four, five, or both, were excluded from the test. Excluded birds were either not interested in the food reward, in which case the reward could not be used as an incentive to complete the test, or they were too afraid to eat in the test pen, in which case they would most likely be too afraid to complete the test. Due to time constraints a maximum number of 15 pullets per pen could be included in the test, if more than 15 pullets met inclusion criteria, surplus pullets were excluded randomly. Excluded birds remained in the home pens to keep stocking density constant. Consequently, 459 pullets qualified for testing (Supplementary Table 4).

Supplementary Table 4. Number of hens participating (met inclusion criteria) in the vertical navigation test by flock, strain, and housing treatment. Qualified pullets from flocks 1 and 2 were further tested in the ramp-choice test.

	Conv	***Low***	***Mid***	***High***	**Total**
**Flock 1**	**20**	**28**	**26**	**30**	**104**
brown	7	13	11	15	46
white	13	15	15	15	58
**Flock 2**	**30**	**30**	**30**	**30**	**120**
brown	15	15	15	15	60
white	15	15	15	15	60
**Flock 3**	**30**	**30**	**30**	**30**	**120**
brown	15	15	15	15	60
white	15	15	15	15	60
**Flock 4**	**28**	**28**	**29**	**30**	**115**
brown	13	15	14	15	57
white	15	13	15	15	58
**Total**	**108**	**116**	**115**	**120**	**459**

Testing. The following week (week 16 of rearing) included five days of testing in a modified version of a spatial test performed by Gunnarsson et al.[5]. Individual testing took place over five days with each pullet being asked to complete a different task every day. The tasks increased in difficulty every day, so that any frustration experienced from failure would not affect performance in an easier task. To successfully complete a task, pullets had to reach the platform with the food reward, which included a handful of sweet corn and 3-5 mealworms. In tasks 1-4, food rewards were placed on the elevated platforms and pullets were placed on the floor, facing away from the reward to minimise the risk of a pullet approaching the reward by accident while fleeing from the experimenter. In task 1, the reward was at 60 cm, in tasks 2 and 3 at 120 cm and in task 4 at 180 cm. For task 5, pullets were placed on the highest platform, facing the wall to minimise the risk of a pullet jumping on the experimenter before they stepped away, with the reward being on the middle platform. Tasks 2 and 4 could be completed with multiple steps, and the lower platforms held reward dishes with only two corn kernels and one mealworm. These lower dishes were meant to draw the pullet's attention upwards, as no assumption about navigation skills could be made if birds were unaware of the reward. Tests were observed live by two observers, one per test pen and success, defined as both feet on the platform, was recorded on paper. Additionally, the quality of each jump attempt was scored on a scale of 1-4 (1= worst, 4=best) where 1 and 2 describe unsuccessful transitions and 3 and 4 successful ones. If no attempt was made within 5 min, the pullet was returned to her home pen without receiving a score. The number of tested birds by housing system and their success per task are detailed in Supplementary Table 4.

Ramp-choice test

Birds from the vertical navigation test in flocks 1 and 2 where then tested in a ramp-choice test (Figure 4). Two pullets were excluded due to injuries and two more pullets were excluded after losing their identifying leg rings. Testing took place over three days with one task per pullet each day where they were given two minutes to complete the task and transition between the ground and the platform either by aerial locomotion, by using the ramp, or by a combination of the two ('mixed' strategy). For task '60U', the red reward dish was placed on the platform at 60cm elevation, and the pullet was placed on the ground facing away from the platform. In task '60D', the reward dish was placed on the ground and the pullet on the platform at 60cm elevation facing the wall. For task '120D', the reward dish was on the ground and the pullet was placed on the platform at 120cm elevation facing the wall. Tasks 60U and 60D were evenly balanced across days one and two while task 120D took place on day three for all pullets to avoid a drop in motivation should that talk be too difficult to complete. Testing was balanced across time of day with half the pullets from a treatment group being tested in the morning and the other in the afternoon. The tests were video recorded (Sony Handycam HDR-X240 and HDR-X110, Tokyo, Japan) and videos were observed by one observer using Noldus Observer XT version 14 (Noldus Information Technology, Wageningen, The Netherlands) in a randomized order generated by an online random number generator (https://www.random.org). Out of the 660 observations, 25 were lost due to a corrupted SD card. The outcome variables recorded were success, latency to succeed, and locomotion strategy of each attempted transition (aerial, ramp, or mixed). Intra-observer reliability was assessed by the observer re-coding 20 randomly chosen videos. The experimenters and the observer were blinded to housing treatment but not to strain, task, or hypothesis.

Supplementary Table 5. Power analysis for the ramp choice test based on the number of degrees of freedom (df) generated and used.

	df		df
**Total**		**Predictors**	
housing	4	housing	3
strain	2	strain	1
flocks	2	task	2
N/ flock/ housing/ strain	10†	housing x strain	3
total df=	160	housing x task	6
**Random**		strain x task	2
housing	4	housing x strain x task	6
strain	2	predictor df =	23
flock	2	**Residual**	
task	3	df tot.- df rand.-intercept-df pred.	
random df=	48	Residual df=	88

† The expected number of pullets to qualify for testing, there ended up being more in every group except for *Conv* browns in flock 1.

Data processing

Hurdle test. Six dependent variables were used to describe chick performance in the hurdle test: success, strategy, latency to walk, vocalisation, and persistence. Success (binary, yes=1, no=0) in the hurdle test was assessed for all tasks combined. Motivation for social reinstatement was assessed by analysing the latency to approach the hurdle, vocalisation frequency (bouts per minute) for all difficulties (1470 observations), and the persistence to cross (number of crossing attempts made) for difficulties 1, 3, and 5 (884 observations) to minimize the risk of overestimating crossing attempts, as chicks occasionally appeared to 'accidentally' walk onto the ramp. For difficulties 2 and 4, when chicks crossed the hurdle, strategy was analysed as a binary

variable with crossing by jumping indicated as 1 and crossing by ramp as 0 (47 observations). Inter-observer reliability was moderate for frequencies (kappa= 0.63) and excellent for durations (kappa= 0.97).

Vertical navigation test. Three dependent variables were used to describe pullet performance in the vertical navigation test: success, jumping quality, and strategy. 'Success' of reaching the final reward was recorded as a binary value per task in all pullets (yes=1 or no=0). For example, in task 2 with two possible jumps (floor to 60 cm and 60 cm/floor to 120cm), the final reward was on the higher platform at 120cm. A pullet that jumped only to the lower platform would have been scored on the quality of that jump but received a zero for success. An independent variable called 'direction' described locomotion required to complete a task as either upwards (tasks 1-4) or downwards (task 5). To analyse the quality of vertical navigation performed, 'jumping quality' scores were averaged over all five tasks. Pullets were only assessed on the jumps attempted, therefore a bird that made no jump attempt would be excluded from this data set. A total of 374 pullets attempted at least one jump.

Ramp-choice test. Three dependent variables were used to assess the performance in the ramp-choice tasks: success, latency to succeed, and strategy. 'Success' was defined as a pullet placing both feet on the vertical target level (platform in 60U, ground in 60D and 120D) and recorded as a binary variable (yes=1 or no= 0, 635 observations). 'Latency to succeed' was defined as the time elapsed between the placement of the pullet and success in seconds, only including observations with successful transitions (399 observations). Strategy was recorded for all attempted transitions (453 observations) as a categorical variable with three levels: 'aerial', 'mixed', or 'ramp'. Intra-observer reliability was excellent (kappa= 0.87).

Statistical analyses

Statistical analyses were done in R and R Studio version 3.5.2 using packages 'lme4', 'car', 'oddsratio, 'emmeans', 'effects', 'gmodels', and 'DHARMa'. Dependent variables in the hurdle test were analysed by fitting generalized-/ linear mixed effect models (G/LMM) with brooding compartment nested in housing style nested in flock as random effect. Successful crossing of the hurdle ("success"), latency to walk and vocalisation frequency were analysed based on the whole data set. To analyse success, a GLMM was fitted with housing, strain, difficulty, and all interactions as fixed effects. Latency to walk and vocalisation frequency were analysed by applying an LMM with housing, strain, and their interaction as fixed effects. For the ramp tests, strategy was analysed for those that crossed (ramp use vs jumping) by applying a GLMM with a binary outcome variable. For the hurdle only tests, persistence was analysed as the total amount of crossing attempts where success was used as one of the independent variables together with housing, strain, and all interactions in a GLMM. Inter-observer reliability was assessed by calculating kappa statistics on Observer (The Observer® XT by Noldus, Version: 14.2) for durations and frequencies.

Jumping quality in the vertical navigation test (374 data points) was analysed by fitting a LMM and the probability of success was analysed with a GLMM. Both models had strain, housing, and their interaction as fixed effects. Direction was included as a fixed effect in the GLMM.

Success in the ramp-choice test (binary, 635 data points) was analysed by fitting a GLMM and latency to succeed (in seconds, 399 data points) was log transformed before an LMM. Both models had housing, strain, and their interaction as fixed effects.

A combination of non-parametric statistics and descriptive statistics were used to analyse locomotion strategy in the ramp-choice test (453 data points).

References

1. NFACC. *Code of Practice for the Care and Handling of Pullets and Laying Hens*. *National Farm Animal Care Council* https://www.nfacc.ca/codes-of-practice/pullets-and-laying-hens (2017).

2. Eisenhauer, J. G. Degrees of freedom. *Teach Stat* **30**, 75–78 (2008).

3. Pandey, S. & Bright, C. L. What Are Degrees of Freedom? *Soc Work Res* **32**, 119–128 (2008).

4. LeBlanc, C., Tobalske, B., Bowley, S. & Harlander-Matauschek, A. Development of locomotion over inclined surfaces in laying hens. *Animal* **12**, 585–596 (2018).

5. Gunnarsson, S., Yngvesson, J., Keeling, L. J. & Forkman, B. Rearing without early access to perches impairs the spatial skills of laying hens. *Appl Anim Behav Sci* **67**, 217–228 (2000).
